# Prostatic stromal sarcoma with neuroectodermal differentiation

**DOI:** 10.1186/1746-1596-7-173

**Published:** 2012-12-07

**Authors:** Hitoshi Yamazaki, Teppei Ohyama, Toshiki Tsuboi, Yoshinori Taoka, Dai Kohguchi, Hiroyoshi Iguchi, Teruaki Ao

**Affiliations:** 1 Department of Pathology, Medical center hospital, Kitasato Institute, Kitasato University, Saitama, Japan; 2 Department of Urology, Medical center hospital, Kitasato Institute, Kitasato University, Saitama, Japan; 3 Department of Radiology, Medical center hospital, Kitasato Institute, Kitasato University, Saitama, Japan

**Keywords:** Prostatic stromal sarcoma, STUMP, Immunohistochemistry, CD99, CD56, Synaptophysin, Neuroectodermal differentiation

## Abstract

**Abstract:**

Prostatic stromal sarcoma is a fairly rare tumor that constitutes approximately 0.1–0.2% of all prostatic cancers. Detailed characteristics of the tumor are still unclear due to its rarity.

We describe a case of prostatic stromal sarcoma in a 63 year-old man who suffered from urinary obstructive symptoms. Palliative transuterine resection was performed and the preliminary histopathological diagnosis was neuroendocrine carcinoma. After chemotherapy, total pelvic exenteration was performed. Histopathologically, the tumor was composed of monotonously proliferating small to medium-sized round cells, which existed in compact islands with loose or dense fibrovascular networks. Immunohistochemically, the tumor cells were widely positive for vimentin, CD56, CD99 and focally positive for synaptophysin, CD10, progesterone receptor, desmin and CD34, but negative for EMA, cytokeratin, estrogen receptor, S-100 and myoglobin. Most of the previously reported tumors exhibited positive stainability for CD10 and progesterone receptor. In addition to these markers, expressions of CD56, CD99 and synaptophysin were characteristically detected in our case. To the best of our knowledge, we present the first case of prostatic stromal sarcoma with characteristic immunohistochemical staining properties. Although the biological characteristics of this rare tumor have not yet been elucidated, these findings suggest prostatic stromal sarcoma can potentially show neuroectodermal differentiation.

**Virtual slide:**

The virtual slide(s) for this article can be found here: http://www.diagnosticpathology.diagnomx.eu/vs/7291874028051262

## Background

Prostatic stromal sarcoma (PSS) is a fairly rare tumor, constituting approximately 0.1% of all prostatic cancers [1,2]. Prostatic sarcoma and related proliferative lesions, including prostatic phyllodes tumors, have been classified as prostatic stromal tumors of uncertain malignant potential (STUMP) and prostatic stromal sarcoma (PSS) based on cellularity, mitotic index, cellular atypia and necrosis (WHO 2004) [3]. Some STUMP cases were reported as malignant transformation into PSS [4]. There has not yet been a clear differentiation between PSS and STUMP due to the rarity of these tumors. In this article, prostatic stromal sarcoma has potentially neuroectodermal characteristics.

## Case presentation

**Figure 1:**
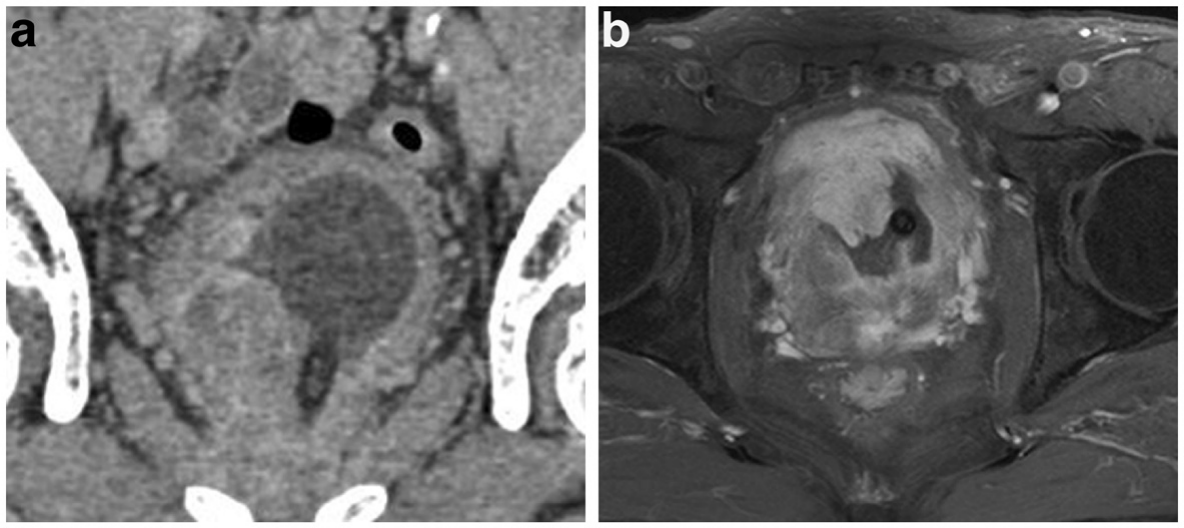
**Image analysis. a**) Computed tomography shows a prostatic non-homogenous mass lesion irregularly protrudingd into the bladder space. **b**) Gadrinium-enhanced T1 weighted magnetic resonance imaging shows a heterogeneous hyperintense mass.

**Figure 2:**
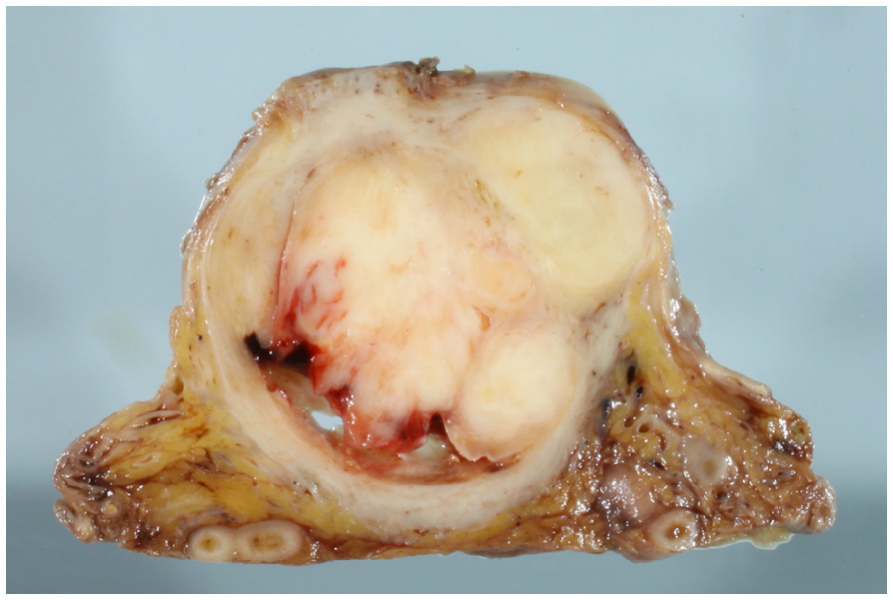
**Cut surface of the tumor.** The prostate and the bladder space are widely occupied by the tumor tissue, which exhibited a whitish-yellow multinodular appearance with focal necrosis.

**Figure 3:**
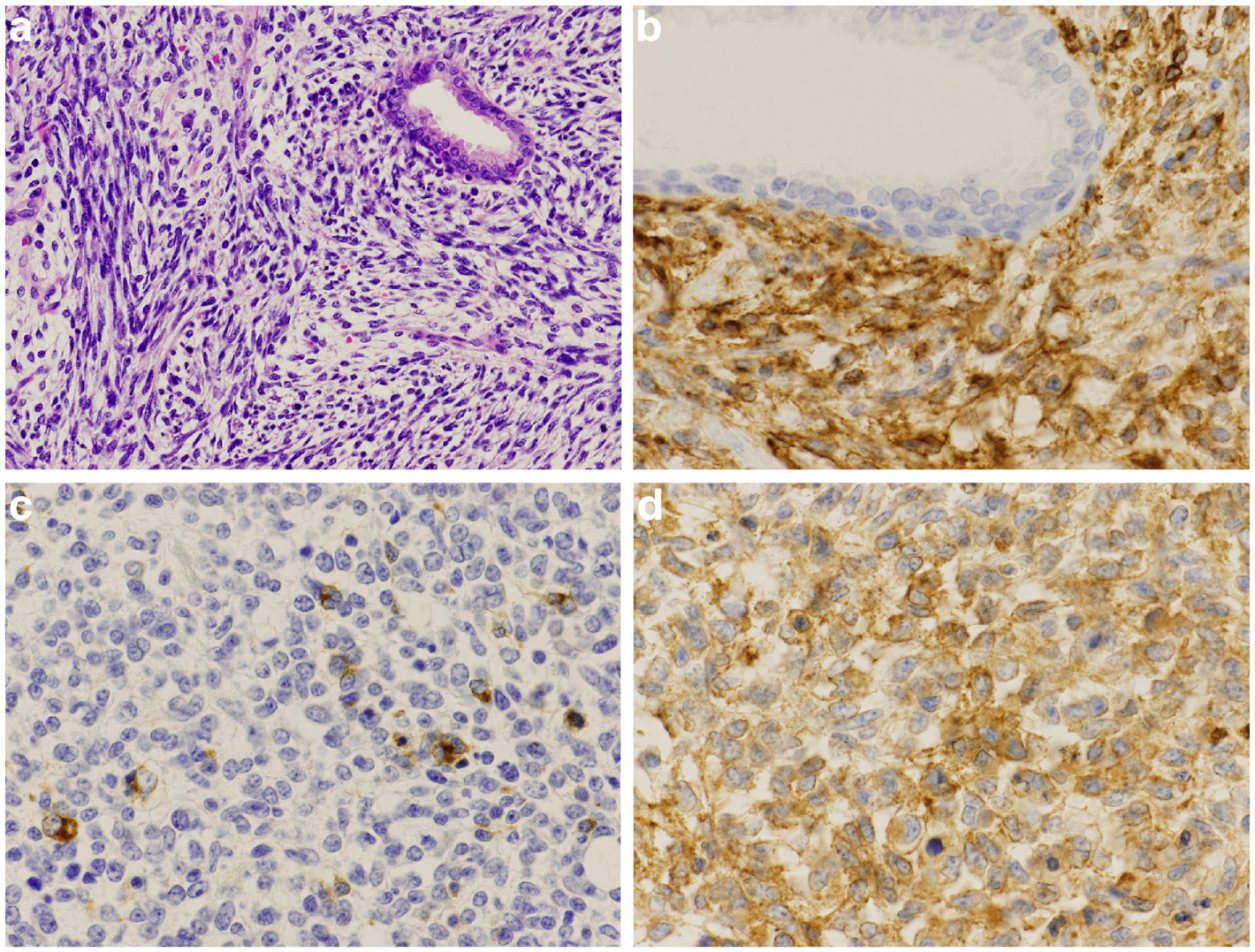
**Microscopic findings. a**)The tumor was made up of sarcomatoid oval to spindle cells. (HE x 100) **b**) Immunostaining with CD56 showed positive results for tumor cells on the cell membrane (CD56 x 200). **c**) Immunostaining with synaptophysin focally showed positive results for the tumor cells in the cytoplasm (synaptophysin x 200). **d**) Immunostaining with CD99 showed positive results for tumor cells on the cell membrane (CD99 x 200).

The patient was a 63 year-old man who presented to the urologist with a one week history of dysuria, pollakiuria and an unrelieved feeling after urination. He had a previous history of diabetes mellitus and asymptomatic multiple brain infarction. He habitually drank alcohol and smoked and his mother died of gastric cancer. Abdominal ultrasonography revealed 343 ml of residual urine volume. Computed tomography revealed a prostatic mass lesion which protruded into the bladder space (Figure 1a). Gadrinium-enhanced T1 weighted magnetic resonance imaging also revealed the prostatic mass lesion had irregularly high signals (Figure 1b). Based on our clinical diagnosis of benign prostatic hypertrophy, palliative transurethral resection was performed. The specimen consisted of 20 grams of piecemealed prostate that was totally embedded in paraffin and histologically analyzed. Sections showed monotonously proliferating small to medium-sized round cells invading the edematous stroma with coarseness and fineness and sparse or dense patterns. The residual prostatic glands were identified. Immunohistochemically, the tumor cells were positive for vimentin, CD56, synaptophysin (focal) but negative for EMA, cytokeratin, S-100. The preliminary histopathological diagnosis to decide the strategy for further therapy was neuroendocrine carcinoma. He took one series of chemotherapy, a combination of cisplatin and irinotecan. Ten days after the last day of the chemotherapy, suprapubital radical cystprostatectomy with rethrotectomy was performed. During the operation, the urinary bladder could be detached from the rectum with difficulty. Finally, low anterior rectal resection was additionally performed, resulting in total pelvic exenteration. When the urethra was cut, a part of the tumor was pressed down from the urethral cavity. The gross features of the tumor were elastic soft and translucently whitish in color. After fixation in 10% formaldehyde the prostate weighed 125 g and contained an ill-defined gray whitish mass lesion, which focally exhibited necrosis (Figure 2). The bladder and the retroperitoneal cavity were diffusely infiltrated by the whitish tumor, which was directly connected to the prostatic tumor. Although the rectum was attached to the tumor mass, the rectal parenchyma was free from tumor invasion. The bladder space was occupied by the tumor and narrowed, resulting in a slit-like space. Serial sections were analyzed and revealed a round cell sarcomatous tumor with occasional higher cellularity than that in the previous transuterine resection. Tumor necrosis was occasionally identified. The N/C ratio of the tumor cells was a relatively high grade. Mitotic activity was measured as 12/10 HPF in the higher cellularity area. The residual prostatic glands were compressed to the periphery (Figure 3a). No lymph nodal metastasis was observed. Immunohistochemically, the tumor cells were widely positive for vimentin, CD56, CD99 and focally positive for synaptophysin, CD10, progesterone receptor, desmin and CD34, but negative for EMA, cytokeratin, estrogen receptor, S-100, GFAP and myoglobin (Figure 3b,c,d). The Ki-67 index was about 70%. We finally diagnosed the tumor as prostatic stromal sarcoma. Sixteen months later, the patient is alive without local recurrence or distant metastasis.

## Discussion

Prostatic stromal sarcoma is a rare tumor that constitutes approximately 0.1–0.2% of all prostatic cancers [1,2]. Prostatic mesenchymal tumors sometimes cannot be clearly classified as histological entities due to their rarity [5]. Except for prostatic mesenchymal tumors with specialized differentiation, a histologically characteristic classification involves prostatic stromal sarcoma and prostatic stromal proliferation of uncertain malignant potential (STUMP) [6-8]. These tumors have been described on the basis of the histological similarity to phyllodes tumors and have to be differentially diagnosed as other specialized types of tumor, such as GIST, leiomyosarcoma, rhabdomyosarcoma(RMS), and fibrosarcoma [3]. RMS is the most common sarcoma of the prostate, but is rarely reported in adult patients [9]. Prostatic stromal sarcoma and STUMP express female hormone receptors, especially progesterone receptor. These tumors seem to be derived from female hormone-dependent stromal cells [10]. Depending on the cellularity, mitosis and necrosis, these two tumors are histologically evaluated. An acceptable histological grading of stromal sarcoma has not yet been proposed due to the rarity of the tumor. Hasegawa reported that the ki-67 index was related to the prognosis of the tumor [4].

Recent immunohistochemical analysis revealed that both prostatic stromal sarcoma and STUMP usually express CD10, CD34 and the progesterone receptor [3,7,8,10-12]. This tumor sometimes positively reacts with smooth muscle actin. However, the detailed histopathological characteristics of the tumor cells have not yet been clearly elucidated. Kim reported a case of prostatic stromal sarcoma with rhabdoid features [13]. The tumor in our case was positive for CD56, CD99 (to our knowledge the second reported case) [14], synaptophysin and negative for EMA and cytokeratin. This is the first description of prostatic stromal sarcoma with immunohistochemically positive stainability for synaptophysin. There are other prostatic non-epithelial malignancies with potential neuroectodermal differentiation such as carcinosarcoma, ectomesenchymoma and primitive neuroectodermal tumor (PNET), which have to be distinguished from our case of prostatic stromal sarcoma. Patient age is a useful marker for differential diagnosis, although PNET and malignant ectomesenchymoma are rarely reported in young adult prostate cancer patients [15-18]. Most PNET cases reveal positive immunoreactivity for CD99, but are negative for progesterone receptor. Carcinosarcoma contains elements of epithelial malignancy which exhibit some types of cytokeratins.

PSS is suspiciously derived from mesenchymal pluripotent stem cells in the prostatic stroma. As few cases of STUMP change to PSS in the history, some genetic transformations are considered to be related to PSS [4,19]. Arva showed that some cases of prostatic squamous cell carcinoma were derived from hormonal or radiation-treated prostatic adenocarcinoma [20]. Unexpected irradiation may be one of the risk factors of PSS [21,22]. Babarović reported a case of high grade angiosarcoma arising in fibroadenoma and suggested the possibility that severe inflammatory reaction, for example silicon granuloma, may cause malignant transformation of stromal cells in the affected area [23,24]. These factors may give rise to an exuberant stromal response and cause some genetic events in the mesenchymal pluripotent stem cells. We could classify PSS as two categories, which are *de novo* tumorigenesis and malignant transformation step by step [20]. However, there are no authentic molecules which directly cause prostatic stromal tumorigenesis. Fibroblast growth factor 8 (FGF8), the eighth member of the fibroblast growth factor family contains alternatively spliced mRNA isoforms. Fibroblast growth factor 8b(FGF8b) is an androgen-induced growth factor with potent oncogenic activity [25-27]. Elo reported the results of prostate-targeted fibroblast growth factor 8b transgenic mice [28]. These mice showed progressive changes in prostatic stroma, as well as the prostatic epithelium. Furthermore, FGF8b is the predominant FGF8 spliceform necessary for proper posterior neural formation in Xenopus [29]. Amsterdam reported the relationship between fgf8 misregulation and neuronal tumors in Zebrafish [30]. This knowledge suggests FBF8b is related to potential neuroectodermal differentiation in prostatic stromal tumorigenesis. Some hormonal imbalances, including androgen imbalance, may cause prostatic stromal progression, which could lead to prostatic stromal neoplasia. We hereby point out that PSS may also possess neuroectodermal characteristics.

## Conclusions

We describe a rare case of prostatic stromal sarcoma (PSS) presenting with characteristic immunohistochemical staining properties. To our knowledge, this case is the first case of PSS with positive stainability for synaptophysin, as well as CD99 and CD56.

### Consent

Written informed consent was obtained from the patient for publication of this case report and accompanying images. A copy of the written consent is available for review by the Editor-in-Chief of Diagnostic Pathology.

## Competing interests

We do not have any competing interests for our manuscript.

## Authors’ contribution

HY was responsible for data collection and drafted the manuscript. TO, TT, YT, DK and TA made contributions to acquisition of clinical data and performed surgical procedure. HI performed radiological interpretation and helped drafting the manuscript. All authors read and approved the final manuscript.

## References

[B1] Bernard T John RS Bostwick DG Bostwick GB Soft tissue tumors Pathology of the Prostate 1990 New York: Churchill Livingstone 117 135

[B2] Bostwick DG Eble JN Bostwick DG, Eble JN Neoplasms of the prostate Urologic Surgical Pathology 1997 Philadelphia: Mosby 399 401

[B3] Cheville J Cheng L Algaba F Boccon-Gibod L Furusato M Billis A Lopez-Beltran A Eble JN, Sauter G, Epstein JI, Sesterhenn IA Mesenchymal tumours Pathology and Genetics of Tumours of the Urinary System and Male Genital Organs 2004 Lyon: IARC Press 209 211 [World Health Organization Classification of Tumours]

[B4] Hasegawa S Yoshikawa M Konomoto T Andou S Inoue M Nagashima A Omiya K Ushijima M Prostatic stromal sarcoma: a case report Nishinihonhinyokika 2002 64 619 626

[B5] Herawi M Epstein JI Specialized stromal tumors of the prostate: a clinicopathologic study of 50 cases Am J Surg Pathol 2006 30 694 704 10.1097/00000478-200606000-00004 16723846

[B6] Gaudin PB Rosai J Epstein JI Sarcomas and related proliferative lesions of specialized prostatic stroma: a clinicopathologic study of 22 cases Am J Surg Pathol 1998 22 148 162 10.1097/00000478-199802000-00002 9500215

[B7] De Berardinis E Busetto GM Antonini G Giovannone R Di Placido M Magliocca FM Di Silverio A Gentile V Incidental prostatic stromal tumor of uncertain malignant potential (STUMP): histopathological and immunohistochemical findings Urologia 2012 79 65 68 10.1016/j.urology.2012.02.008 22388992

[B8] Brolin J Skoog L Ekman P Immunohistochemistry and biochemistry in detection of androgen, progesterone, and estrogen receptors in benign and malignant human prostatic tissue Prostate 1992 20 281 295 10.1002/pros.2990200404 1376911

[B9] Waring PM Newland RC Prostatic embryonal rhabdomyosarcoma in adults. A clinicopathologic review Cancer 1992 69 755 762 10.1002/1097-0142(19920201)69:3<755::AID-CNCR2820690324>3.0.CO;2-Y 1730126

[B10] Stoll LM Johnson MW Rosenthal DL High-grade prostatic sarcoma seen in a catheterized urine specimen: case report and differential diagnosis Diagn Cytopathol 2011 39 762 766 10.1002/dc.21545 20890999

[B11] Colombo P Ceresoli GL Boiocchi L Taverna G Grizzi F Bertuzzi A Santoro A Roncalli M Prostatic stromal tumor with fatal outcome in a young man: histopathological and immunohistochemical case presentation Rare Tumors 2010 31;2 4 e57 21234249 10.4081/rt.2010.e57 PMC3019592

[B12] Osaki M Osaki M Takahashi C Miyagawa T Adachi H Ito H Prostatic stromal sarcoma: case report and review of the literature Pathol Int 2003 53 407 411 10.1046/j.1440-1827.2003.01489.x 12787317

[B13] Kim JY Cho YM Ro JY Prostatic stromal sarcoma with rhabdoid features Ann Diagn Pathol 2010 14 453 456 10.1016/j.anndiagpath.2009.10.008 21074696

[B14] Segawa N Hamada S Takahara K Azuma H Tsuji M Katsuoka Y Prostatic stromal sarcoma: a case report Hinyokika Kiyo 2008 54 29 34 18260357

[B15] Kumar V Khurana N Rathi AK Malhotra A Sharma K Abhishek A Bahadur AK Primitive neuroectodermal tumor of prostate Indian J Pathol Microbiol 2008 51 386 388 10.4103/0377-4929.42518 18723965

[B16] Funahashi Y Yoshino Y Hattori R Ewing’s sarcoma/primitive neuroectodermal tumor of the prostate Int J Urol 2009 16 769 19769660 10.1111/j.1442-2042.2009.02339.x

[B17] Paramelle O Croué A Dupré F Rialland X Saint-André JP Pelvic malignant ectomesenchymoma: a case report Ann Pathol 2001 21 344 347 11685134

[B18] Colecchia M Dagrada G Poliani PL Messina A Pilotti S Primary primitive peripheral neuroectodermal tumor of the prostate. Immunophenotypic and molecular study of a case Arch Pathol Lab Med 2003 127 e190 193 12683899 10.5858/2003-127-e190-PPPNTO

[B19] Watanabe M Yamada Y Kato H Imai H Nakano H Araki T Shiraishi T Malignant phyllodes tumor of the prostate: retrospective review of specimens obtained by sequential transurethral resection Pathol Int 2002 52 777 783 10.1046/j.1440-1827.2002.01417.x 12588447

[B20] Arva NC Das K Diagnostic dilemmas of squamous differentiation in prostate carcinoma case report and review of the literature Diagn Pathol 2011 6 46 10.1186/1746-1596-6-46 21627811 PMC3118316

[B21] Hossain D Meiers I Qian J MacLennan GT Bostwick DG Prostatic stromal hyperplasia with atypia: follow-up study of 18 cases Arch Pathol Lab Med 2008 132 1729 1733 18976007 10.5858/132.11.1729

[B22] Bostwick DG Egbert BM Fajardo LF Radiation injury of the normal and neoplastic prostate Am J Surg Pathol 1982 6 541 551 10.1097/00000478-198209000-00006 7149094

[B23] Babarović E Zamolo G Mustać E Strčić M High grade angiosarcoma arising in fibroadenoma Diagn Pathol 2011 6 125 10.1186/1746-1596-6-125 22185665 PMC3284406

[B24] Takenaka M Tanaka M Isobe M Yamaguchi R Kojiro M Shirouzu K Angiosarcoma of the Breast with Silicone Granuloma: A Case Report Kurume Med J 2009 56 33 37 10.2739/kurumemedj.56.33 20103999

[B25] Tanaka A Miyamoto K Minamino N Takeda M Sato B Matsuo H Matsumoto K Cloning and characterization of an androgen-induced growth factor essential for the androgen-dependent growth of mouse mammary carcinoma cells Proc Natl Acad Sci USA 1992 89 8928 8932 10.1073/pnas.89.19.8928 1409588 PMC50037

[B26] Ghosh AK Shankar DB Shackleford GM Wu K T'Ang A Miller GJ Zheng J Roy-Burman P Molecular cloning and characterization of human FGF8 alternative messenger RNA forms Cell Growth & Differentiation 1996 7 1425 1434 8891346

[B27] Kouhara H Koga M Kasayama S Tanaka A Kishimoto T Sato B Transforming activity of a newly cloned androgen-induced growth factor Oncogene 1994 9 455 462 8290257

[B28] Elo TD Valve EM Seppänen JA Vuorikoski HJ Mäkelä SI Poutanen M Kujala PM Härkönen PL Stromal activation associated with development of prostate cancer in prostate-targeted fibroblast growth factor 8b transgenic mice Neoplasia 2010 12 915 927 21076617 10.1593/neo.10776 PMC2978914

[B29] Fletcher RB Baker JC Harland RM FGF8 spliceforms mediate early mesoderm and posterior neural tissue formation in Xenopus Development 2006 133 1703 1714 10.1242/dev.02342 16554360

[B30] Amsterdam A Lai K Komisarczuk AZ Becker TS Bronson RT Hopkins N Lees JA Zebrafish Hagoromo mutants up-regulate fgf8 postembryonically and develop neuroblastoma Mol Cancer Res 2009 7 841 850 10.1158/1541-7786.MCR-08-0555 19531571 PMC2744123

